# Development and implementation of a HAPA-based exercise rehabilitation program for patients with chronic heart failure

**DOI:** 10.3389/fcvm.2026.1801286

**Published:** 2026-05-04

**Authors:** Jiang-Ying Li, Qiu-Chen Wang, Lu Chen, Li-Chun Wang, Li Huang

**Affiliations:** Department of Nursing, The Affiliated Taizhou People’s Hospital of Nanjing Medical University, Taizhou School of Clinical Medicine, Nanjing Medical University, Taizhou, China

**Keywords:** chronic heart failure, exercise adherence, exercise rehabilitation, health action process approach model, self-efficacy

## Abstract

**Objective:**

To develop a Health Action Process Approach (HAPA) based exercise rehabilitation program for chronic heart failure (CHF) patients and evaluate its effectiveness.

**Methods:**

A randomized controlled trial was carried out in which 100 patients with CHF admitted to the Department of Cardiology between April 2024 and April 2025 were recruited. Participants were randomly allocated to either the control group (*n* = 50) receiving routine care or the intervention group (*n* = 50) receiving the HAPA-based exercise rehabilitation program for three months. Primary outcomes were exercise adherence, exercise self-efficacy and quality of life (QoL), which were compared between the two groups.

**Results:**

During the study, three and two patients in the intervention and control groups, respectively, withdrew, leaving 95 people for final analysis. Exercise adherence scores showed significant time and group effects (both *P* < 0.001), but the time by group interaction was not statistically significant (*P* = 0.141). No substantial between-group variances were found at baseline (*P* = 0.070), however, the intervention group had significantly higher exercise adherence scores at 1, 3 and 6 months post-intervention (all *P* < 0.001). At three months, exercise self-efficacy was significantly greater in the intervention group (MD = 7.57, 95% CI: 3.98–11.17; Cohen's d = 0.86; *P* < 0.001). Additionally, total QoL scores as well as scores in the physical, emotional, and other domains were substantially lower in the intervention group relative to the control group (all *P* < 0.001), suggesting improved QoL.

**Conclusion:**

Exercise rehabilitation program based on HAPA may improve exercise adherence, exercise self-efficacy and QoL in patients with chronic heart failure. Nevertheless, additional studies are needed to verify its efficacy and its broader clinical usefulness.

## 1 Introduction

Chronic heart failure (CHF) is a severe complication of many cardiovascular diseases, which is the end stage of the disease process and a major cause of cardiovascular mortality (1). Worldwide, there are around 40 million people who suffer from heart failure (2). Patients with CHF usually develop symptoms such as low tolerance to exercise and dyspnea, which is often accompanied by negative emotions (i.e., depression and anxiety) (3). Studies show that exercise rehabilitation can be a very effective method for improving exercise capacity and cardiopulmonary function, decreasing hospitalization and mortality rates, and slowing the progression of the disease in CHF patients (4); Consequently, exercise rehabilitation is a Class I recommendation in domestic and international guidelines (1, 5). Despite these benefits, exercise adherence among CHF patients remains universally poor and implementation of exercise rehabilitation programs is suboptimal (6). Studies suggest that participation rates in heart failure patients eligible for exercise rehabilitation are less than 20% (7), thus there is an urgent need for effective interventions to improve adherence. The Health Action Process Approach (HAPA) is a comprehensive theoretical model that separates the behavior change process into three phases: pre-intention, intention, and action phases, with a focus on the role of stage-specific interventions in promoting behavior change (8). In the pre-intention phase, individuals have not yet formed a clear intention to change, but motivation is triggered by enhancing psychological factors such as action self-efficacy, outcome expectations and risk perception. The intention phase concerns the willingness of individuals to change, where they would make specific action plans and coping strategies, which would increase the maintenance self-efficacy and lead to the transformation of intention into behavior. The action phase includes the initiation and maintenance of healthy behaviors where a key focus is on self-efficacy restoration to help people cope with setbacks and persevere in healthy behaviors. The HAPA model therefore gives a theoretical basis for behaviour change in clinical nursing, with stage-specific intervention being effective for stimulating the shift from intention to action and facilitating the maintenance of healthy behaviours (9). Currently, the HAPA model has been broadly utilized in different fields of health behaviors and has shown great achievements (10–12). McCleary et al. found a HAPA-based intervention to significantly improve completion rate of cardiac rehabilitation after myocardial infarction (13). Similarly, Aliabad et al. used HAPA-based interventions in conjunction with family support for coronary artery disease patients undergoing cardiac rehabilitation, which improved family support and the ability of patients to continue physical activity post hospital discharge (14). Based on this evidence, the current study is planned to design an exercise rehabilitation program for CHF patients on the basis of the HAPA model and assess the application effect, and it is expected to provide theoretical basis and practical reference for clinical medical staff implementing exercise rehabilitation.

## 2 Materials and methods

### 2.1 Clinical data

**Figure 1:**
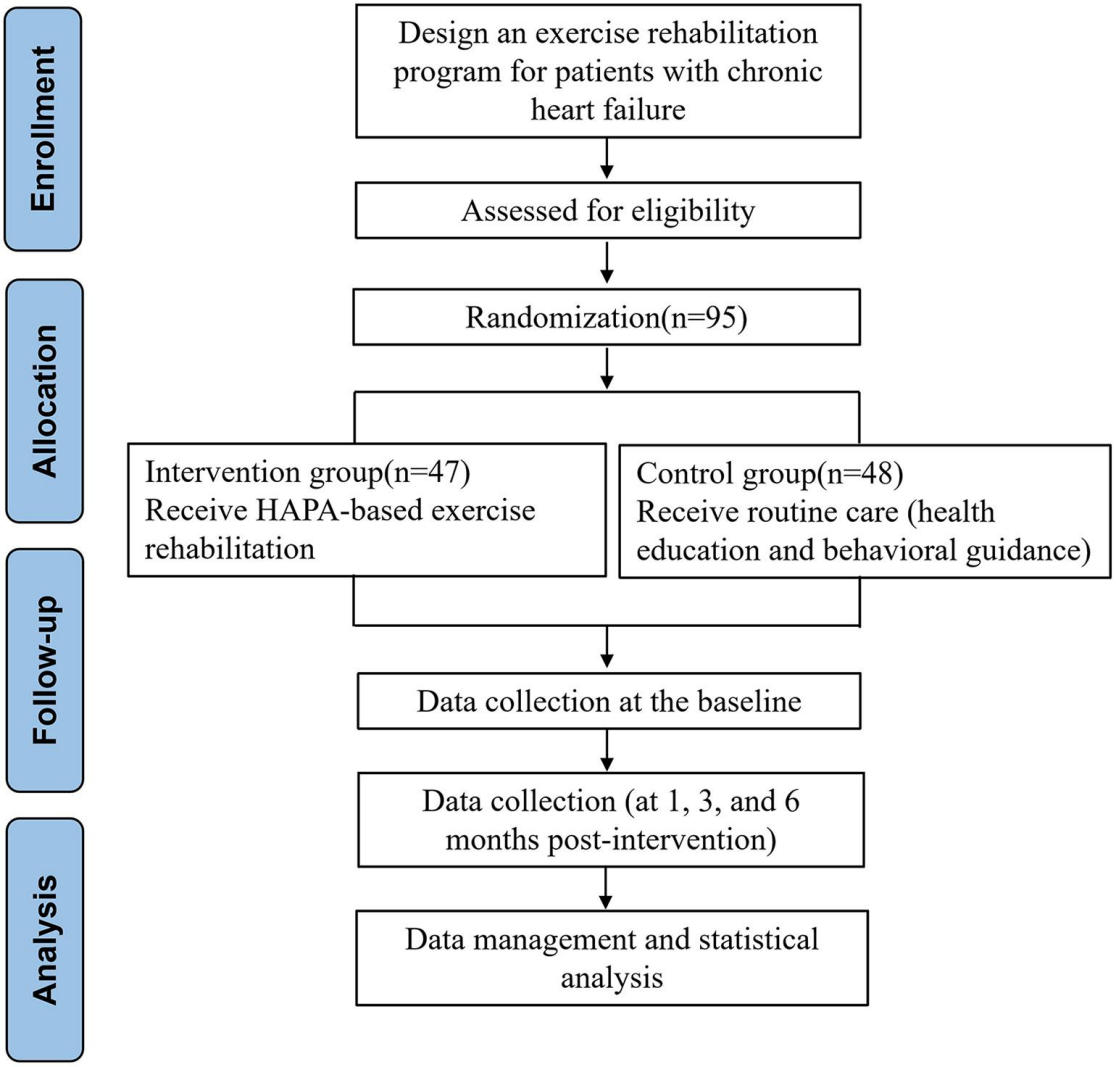
Flow chart of the study design.

Utilizing convenience sampling, 100 patients with CHF admitted to the Department of Cardiovascular Medicine, our hospital between April 2024 and April 2025 were recruited as study participants. Patients were randomly allocated to control or intervention group utilizing a random number table. 50 patients were allocated to each group. Inclusion Criteria: (1) Age 18 to 75 years; (2) Definitely diagnosed with CHF according to 2018 Chinese Guidelines for the Diagnosis and Treatment of Heart Failure (15); (3) New York Heart Association (NYHA) functional class II or III; (4) Patient or primary caregiver is well-versed in the use of WeChat; (5) Provided informed consent and voluntarily agreed to participate. Exclusion Criteria: (1) Associated mental disorders that could interfere with communication; (2) Severe comorbidities, such as malignant tumors, liver or kidney failure or severe arrhythmias; (3) Inability to engage in exercise rehabilitation. This study was approved by the Hospital Ethics Committee (KY2024-079-01), and all participants gave written informed consent. The flowchart of the study design is given in Figure 1.

### 2.2 Randomization, allocation concealment and blinding

Eligible patients were allocated to the control or intervention group in a 1:1 ratio utilizing a random number table generated in the software package, Statistical Package and Software Corporation (SSP) 25.0. The randomization sequence was generated and managed by an independent researcher not participated in outcome assessment, intervention delivery or participant recruitment. Allocation was hidden in opaque, sealed, and sequentially numbered envelopes, which were opened by the study coordinator only after completion of baseline data collection and confirmation of eligibility. Due to the nature of the intervention, the blinding of participants and providers of the intervention was not possible. However, personnel involved in data collection, data entry and statistical analysis were blinded to group assignments in order to reduce selection and measurement bias.

### 2.3 Intervention methods

#### 2.3.1 Control group

The control group received routine care that included health education and behavioral guidance and psychological counseling. The content of care included: 1) Education on CHF related knowledge, dietary advice, medication instructions, and activity recommendations to improve patient's knowledge of disease management; 2) Help in identifying clinical signs of acute episode and possible triggers; 3) Regular psychological counseling to help with issues such as anxiety and depression, helping patients to maintain a positive mindset. Each patient in the control group had at least two sessions per week, with each session taking about 30 min, for a total of 12 weeks. This approach ensured that patients received standard care and support but did not include a structured exercise rehabilitation program or HAPA-based interventions.

#### 2.3.2 Intervention group

On the basis of the intervention of the control group, an exercise rehabilitation protocol based on the HAPA model was implemented (see Table 1 for details).

1. Establishment of the Intervention Team: The intervention team consisted of seven members: one cardiologist, one rehabilitation physician, one head nurse and four nurses, who were responsible for the design and implementation of the intervention protocol. All team members held at least a bachelor's degree, had more than five years of experience in the field of cardiology or rehabilitation, and had a good working knowledge of CHF-related nursing. Prior to the start of the intervention, the whole team received systematic training to ensure they had the professional knowledge and skills to deliver the program effectively.
2. Construction of the Intervention Protocol: Relevant literature was retrieved from Chinese and English databases, guideline networks and professional association websites using keywords such as “Heart Failure/CHF” and “HAPA” (search terms for English literature were “heart failure,” “heart disease,” “cardiopathy,” and “ HAPA.” Effective intervention strategies were extracted to help develop a preliminary draft of the exercise rehabilitation protocol. The preliminary program was then refined through expert consultation by means of a brainstorming approach. Experts invited to review the protocol had at least a bachelor's degree, a senior professional title or higher and at least 10 years of experience in cardiovascular disease, exercise rehabilitation or nursing. They evaluated the scientific validity, effectiveness, feasibility and practicality of the intervention content and the program was modified based on their feedback. Subsequently, a pilot test was carried out with 10 patients with CHF that met the inclusion and exclusion criteria to assess the safety, feasibility, and acceptability of the program. Findings from the pilot study were then used to further optimize both the intervention content and implementation procedures, leading to the final HAPA based exercise rehabilitation protocol.
3. Intervention content: The intervention was guided by the HAPA model and was divided into three stages, pre-intentional, intentional and action, with stage specific strategies tailored to the behavioral status of patients. Pre-Intentional Phase: At this phase, patients have not yet made an intention to engage in exercise rehabilitation. The most important objective is to get people motivated to exercise. Strategies included: 1) Delivering face-to-face lectures, video presentations, and educational materials to patients to help them understand the etiology, clinical manifestations, treatment strategies, and potential complications of heart failure, which will increase the awareness of the disease and emphasize the seriousness of the disease. 2) Explaining the benefits of long-term exercise for cardiac rehabilitation to patients to increase their understanding of the positive effects of physical activity on health. 3) Inviting patients with good rehabilitation results to share their experiences with the patients to increase their confidence and motivation for exercise. Intentional Phase: Patients in this phase are willing to exercise but have not actually started and have not made a concrete plan. The aim is to aid in the formulation of an individualized exercise plan to ensure actionable steps. Strategies included: 1) Performing a thorough exercise risk evaluation and, depending on the patient's condition, physical status and personal preferences, developing an individualized exercise prescription: Mode: Focus on patient-preferred aerobic exercises, such as walking, jogging, cycling, Tai Chi, or Baduanjin. Frequency: Slowly progress from 2 to 3 sessions a week to 3–5 sessions a week, depending on patient tolerance. Intensity: Start with lower intensity and slowly raise to moderate to high intensity. The goal is mild sweating and moderately increased breathing without tightness in the chest or difficulty breathing. Heart rate should be no more than 70%–80% of the maximum (HRmax = 220—age). Duration: Each session includes 5–10 min of warm up, 10–45 min of formal exercise and 10 min of cool down. For patients who cannot tolerate constant exercise, intermittent exercise is permitted to progress to target intensity. 2) Educating patients and family members about precautions to take while exercising, monitoring methods, and family participation in the rehabilitation process. Action Phase: At this point, patients have begun and continue to engage in regular exercise. The main focus is to assure efficacy and adherence, and support for sustained engagement. Strategies included: 1)Establishing an Exercise Rehabilitation WeChat Group, a platform for regular sharing of rehabilitation videos, patient and family communication, and daily exercise check-in supervision. 2) Exercise logbooks were distributed before discharge, containing instructions for patients and family members to record the frequency, mode, and intensity of exercise. Participants were required to upload photos to the WeChat group after each session and medical staff provided regular guidance. 3)Conducting biweekly assessments of exercise rehabilitation progress, providing timely positive feedback, encouragement and adjustments as needed to address barriers and enhance adherence. 4) Emphasizing maintenance of the target intensity range. Patients who were exceeding the target were taught self-monitoring and progressive reduction in intensity of exercise. Exercise was to be stopped immediately if symptoms such as chest pain, dizziness or palpitations occurred, with prompt medical evaluation as necessary.
4. Implementation of the intervention: The 12 week intervention was implemented by the research team in a standardised fashion. During the hospitalization, patients underwent three intervention sessions, one group-based and two individualized, with a duration of about 30 min each. These sessions included face to face health education, exercise risk assessment, and an individualized exercise prescription. Prior to discharge, patients were taught how to complete the exercise log and use the WeChat group. Whenever feasible, patients and primary caregivers were encouraged to participate jointly so that the intervention would be more effective. After the discharge, the intervention continued mainly through the WeChat group, with materials being delivered weekly. Researchers routinely monitored exercise records that were uploaded by patients and returned timely feedback. Exercise rehabilitation outcomes were evaluated every 2 weeks, and exercise prescriptions were modified based on patients' tolerance and adherence. To ensure fidelity and consistency of intervention across providers, all interventions were implemented by uniformly trained research staff according to a predefined protocol. Standardization was ensured by the use of consistent educational content, structured follow-up procedures, uniform requirements for completing exercise logs, and standardized methods of feedback. Additionally, the research team conducted regular meetings to review the implementation process and reduce the potential amount of variation among providers.

**Table 1:** HAPA-based exercise rehabilitation program for patients with CHF.

Stage	Strategy	Measures
Pre-intentional Phase	Risk perception	Through face-to-face lectures, video playback, and distribution of educational materials, help patients fully understand the etiology, clinical manifestations, treatment points, and complications of heart failure, thereby enhancing disease cognition and helping them recognize the gravity of the condition.
	Outcome expectancies	Explain in detail the positive effects of exercise on cardiac rehabilitation to help patients understand the benefits of long-term exercise for health.
	Self-efficacy	Invite patients with significant rehabilitation results to share successful experiences to boost confidence in exercise.
Intentional Phase	Goal setting and planning	1) Conduct a comprehensive exercise risk assessment. Combined with the patient's condition, physical status, and personal preferences, formulate an individualized exercise prescription. Details include: a. Mode: Prioritize aerobic exercises preferred by the patient, such as walking, jogging, cycling, Tai Chi, or Baduanjin.b. Frequency: Gradually increase from 2 to 3 times per week to 3–5 times per week, with adjustments made flexibly based on patient tolerance. c. Intensity: Start from low intensity and gradually transition to moderate-to-high intensity. The target is for the patient to sweat slightly and have slightly accelerated breathing without chest tightness or shortness of breath. The heart rate should not exceed 70%–80% of the maximum heart rate (HRmax = 220—age). d. Duration: Each session includes 5–10 min of warm-up, 10–45 min of formal exercise, and 10 min of cool-down. If the patient cannot tolerate continuous exercise, intermittent exercise may be used to gradually reach the target intensity. 2) Explain exercise precautions to patients and their families, assist family members in understanding exercise monitoring methods, and encourage their participation in the rehabilitation process.
Action Phase	Action, maintenance, and recovery self-efficacy	1) Establish an Exercise Rehabilitation WeChat Group to regularly push rehabilitation videos, facilitate learning and communication among patients and families, and supervise daily exercise check-ins. 2) Distribute exercise logbooks prior to discharge. Instruct patients and families to record the frequency, mode, and intensity of exercise. Require them to upload photos to the WeChat group after each session, with medical staff answering questions regularly. 3) Conduct an exercise rehabilitation effect assessment every two weeks. Provide timely positive encouragement and feedback to help enhance confidence in continuing exercise. For failure to exercise as planned, analyze the reasons and provide appropriate adjustment suggestions to help overcome difficulties and ensure efficacy. 4) Emphasize maintaining the target intensity range during exercise. If the target is exceeded, strengthen self-monitoring and gradually slow the exercise rate. If symptoms such as chest pain, dizziness, or palpitations occur, exercise must stop immediately, and timely treatment should be sought.

### 2.4 Evaluation indicators

#### 2.4.1 Exercise adherence

This indicator was determined using the Exercise Adherence Scale for Patients with CHF, developed by Gao Min et al. in 2023 (16). The scale has been illustrated to be a valid and reliable measure, with a Cronbach's *α* coefficient of 0.905 and a content validity index of 0.93. It consists of 11 items in two dimensions, prescription adherence (5 items) and monitoring adherence (6 items). Responses are scored on a Likert scale of 5 (1 = “never,” 5 = “always”), with a possible score range of 11–55, with higher scores reflecting greater adherence to exercise.

#### 2.4.2 Exercise self-efficacy

This indicator was determined utilizing the Exercise Self-Efficacy Scale developed by Li Dan et al. in 2008 (17). The scale contains 15 items in 4 dimensions: physical factors, psychological factors, physical environmental factors, and social environmental factors. Items are scored on a 5-point Likert scale from 1 (“completely unconfident”) to 5 (“completely confident”), with higher scores indicating more self-efficacy. The scale has excellent reliability with an overall Cronbach's *α* of 0.92.

#### 2.4.3 Quality of Life (QoL)

QoL was assessed utilizing the Minnesota Living with Heart Failure Questionnaire (MLHFQ). The Chinese version, validated by Zhu Yanbo et al. (18), consists of 21 items in physical (8 items), emotional (5 items) and other domains (8 items). Each item is scored on a scale from 0 to 5, giving a total score range of 0–105, with higher scores indicating poorer quality of life. The questionnaire has been found to show good reliability, with a Cronbach's *α* of 0.88.

### 2.5 Data collection methods

Baseline data were gathered by face-to-face interview between researchers and patients before the intervention. Given that the intervention lasted three months, exercise self-efficacy and QoL were used to determine changes in the psychological-behavioral status and the overall health of the patients, and were therefore measured at baseline and at three months post-intervention. In contrast, adherence to exercise was measured at baseline and 1, 3, and 6 months after the intervention to determine maintenance of exercise behavior during and after the intervention period. Post-intervention data were obtained by telephone follow-ups at 1, 3, and 6 months. Data collection was undertaken independently by two trained researchers using a double-entry and double-verification method to ensure accuracy and reliability.

### 2.6 Statistical analysis

All statistical analyses were carried out with the aid of the statistical software of the International Bureau of Statistics (IBS)—Statistical Package for Social Sciences (SPSS) 24.0. Continuous variables that followed a normal distribution were reported as mean ± standard deviation $\left(\bar{x}\pm s\right)$ and the difference between groups was compared using the independent samples *t*-test. Non-normally distributed continuous variables were expressed as median (P_25_, P_75_) and compared by the rank-sum test. Repeated-measures analysis of variance (ANOVA) was used to assess change over multiple time points. Categorical data were presented as percentages and frequencies and were compared using the Chi-square (*χ*^2^) test. A two-sided *P*-value of < 0.05 was considered statistically significant.

## 3 Results

### 3.1 Comparison of general data between the Two groups

During the study, one patient in the intervention group was lost to follow-up because of disease exacerbation and two were lost to follow-up. In the control group, two patients were lost to follow-up. As a result, 95 participants were included in the final analysis (47 in the intervention group and 48 in the control group). There were no statistically substantial differences between the two groups with regard to demographic and clinical characteristics at baseline (all *P* > 0.05) (Table 2).

**Table 2:** Comparison of general data between the Two groups.

Variables	Intervention group (*n* = 47)	Control Group (*n* = 48)	Statistic	*P*
Age (years, $\bar{x}\pm s$)	61.70 ± 12.11	63.40 ± 12.55	−0.67^a^	0.51
Gender (n)			0.59	0.44
Male	31	28		
Female	16	20		
Marital Status (n)			0.57^b^	0.45
Unmarried	2	5		
Married	45	43		
Educational Level (n)			0.18^c^	0.95
Primary school or lower	22	24		
Middle/High school	21	20		
Junior college or higher	4	4		
Average Monthly Income (n)			2.57	0.28
≤3,000	27	35		
3,000∼5,000	13	9		
≥5,000	7	4		
Smoking History (n)			1.05	0.31
Yes	13	18		
No	34	30		
Drinking History (n)			0.74	0.39
Yes	11	15		
No	36	33		
Medical Payment Method (n)			2.91^b^	0.09
Medical insurance	39	46		
Self-pay	8	2		
NYHA Functional Class (n)			0.97	0.33
Class II	15	11		
Class Ⅲ	32	37		
Disease Duration (n)			4.63	0.10
<1 year	16	13		
1–5 years	21	15		
>5 years	10	20		

In the statistics column,.

a denotes the *t*-value,.

b denotes the continuity-corrected *χ*^2^ test,.

c denotes Fisher's exact test, and the remainder denote the Pearson *χ*^2^ test.

### 3.2 Comparison of exercise adherence scores between the Two groups

Repeated-measures ANOVA suggested substantial time's main effects and group on exercise adherence scores (*F* = 20.889 and *F* = 52.798, respectively; both *P* < 0.001), and the time by group interaction was not statistically significant (*F* = 1.866, *P* = 0.141). Between-group comparisons revealed no significant variance in the adherence to exercise at baseline (*P* = 0.070). However, at 1, 3, and 6 months after intervention, the intervention group had substantially higher exercise adherence scores than the control group (all *P* < 0.001) (Table 3).

**Table 3:** Comparison of exercise adherence scores between the Two groups before and after intervention (scores, $\bar{x}\pm s$).

Time point	Intervention group (*n* = 47)	Control Group（*n* = 48)	*t*	*P* value
Pre-intervention	27.30 ± 8.14	24.17 ± 8.15	1.81	0.07
1 Month Post-intervention	31.83 ± 6.00	26.06 ± 7.20	4.24	<0.001
3 Months Post-intervention	36.66 ± 5.13	29.42 ± 6.04	6.29	<0.001
6 Months Post-intervention	35.11 ± 6.27	27.35 ± 5.09	6.63	<0.001
Time effect	*F* = 20.889, *P* < 0.001			
Group effect	*F* = 52.798, *P* < 0.001			
Time × group interaction	*F* = 1.866, *P* = 0.141			

### 3.3 Comparison of exercise self-efficacy scores between the Two groups

At baseline, there was no substantial variance between exercise self-efficacy scores of the intervention and control groups (MD = 1.59, 95% CI: −2.00 to 5.18; Cohen's d = 0.18; *P* = 0.38). Following the intervention, the intervention group had suggestively higher exercise self-efficacy scores than the control group (MD = 7.57, 95% CI: 3.98 to 11.17; Cohen's d = 0.86; *P* < 0.001) (Table 4).

**Table 4:** Comparison of exercise self-efficacy scores between the Two groups before and after intervention $\left(\mathrm{scores},\bar{x}\pm s\right)$.

Group	*n*	Exercise Self-Efficacy	
		Pre-intervention	3 Months Post-intervention
Intervention group	47	32.28 ± 8.13	40.47 ± 9.64
Control Group	48	30.69 ± 9.43	32.90 ± 7.94
*t*		0.88	4.18
*P*		0.38	<0.001
MD (95% CI)		1.59(−2.00 to 5.18)	7.57 (3.98 to 11.17)
Cohen's d		0.18	0.86

### 3.4 Comparison of QoL scores between the Two groups

At baseline, no significant variances between intervention and control groups were found in total QoL scores or individual domains such as physical, emotional, and other domains (all *P* > 0.05). At 3 months after intervention, the intervention group had substantially lower scores indicating improved QoL when compared with the control group across all domains and the total score (all *P* < 0.001). Specifically, the between group mean differences at three months were: Physical domain: MD = −5.06, 95% CI: −7.69 to −2.41; Cohen's d = −0.779 Emotional domain: MD = −3.98, 95% CI: −5.96 to −1.99; Cohen's d = −0.817 Other domains: MD = −6.63, 95% CI: −9.23 to −4.03; Cohen's d = −1.039 (Table 5).

**Table 5:** Comparison of QoL scores between the Two groups before and after intervention $\left(\mathrm{scores},\bar{x}\pm s\right)$.

Group	*n*	Physical Domain		Emotional Domain		Other Domains		Total Score	
		Pre	3 Mos	Pre	3 Mos	Pre	3 Mos	Pre	3 Mos
Intervention	47	30.34 ± 6.07	21.57 ± 6.48	17.23 ± 4.44	10.19 ± 5.25	27.81 ± 8.21	17.77 ± 6.26	75.38 ± 14.32	49.53 ± 11.42
Control	48	29.00 ± 5.59	26.63 ± 6.49	16.85 ± 4.82	14.17 ± 4.46	28.79 ± 6.13	24.40 ± 6.50	74.65 ± 11.67	65.19 ± 12.56
*t*		1.12	−3.80	0.40	−3.98	−0.66	−5.06	0.28	−6.35
*P*		0.26	<0.001	0.69	<0.001	0.51	<0.001	0.78	<0.001
MD (95% CI)		1.34 (−1.04 to 3.72)	−5.06 (−7.69 to −2.41)	0.38 (−1.51 to 2.27)	−3.98 (−5.96 to −1.99)	−0.98 (−3.93 to 1.96)	−6.63 (−9.23 to −4.03)	0.73 (−4.58 to 6.05)	−15.66 (−20.55 to −10.76)
Cohen's d		0.230	−0.779	0.082	−0.817	−0.136	−1.039	0.057	−1.303

## 4 Discussion

### 4.1 Impact of HAPA-based exercise rehabilitation on exercise adherence

The findings of this study suggest that the HAPA-based exercise rehabilitation program may be effective in improving the level of exercise adherence in patients with CHF. Evidence suggests that the effectiveness of exercise rehabilitation is strongly associated with adherence and good adherence is critical for achieving meaningful clinical outcomes (19). However, previous studies report that CHF patients usually have low levels of exercise knowledge and engagement, with more than 60% failing to meet basic daily exercise requirements, and some not exercising at all (20). To address this issue the present study implemented a staged intervention based on the HAPA model. Adherence scores of exercise in the intervention group were substantially greater than the control group at 1, 3, and 6 months post-intervention (all *P* < 0.001), which implies that the program is effective in promoting adherence. During the motivational phase, interventions in health education, individualized goals, and risk perception probably helped patients to gain a better understanding of exercise rehabilitation, which could help them make the transition from the “pre-intentional” to the “intentional” stage. One month after the intervention, the level of exercise adherence in the intervention group was significantly higher than that in the control group (*P* < 0.001), suggesting that motivational phase interventions were effective in stimulating behavioral intentions. In the action phase, individualized exercise prescriptions, stage-specific feedback, and enhanced social support were likely to have been important in producing the sustained higher adherence at 3 and 6 months (*P* < 0.001), indicating the potential of the HAPA-based approach to sustain exercise behavior over time. However, while repeated measures ANOVA indicated significant time and group effects (*P* < 0.001), the time by group interaction was not statistically significant (*P* = 0.141), suggesting that although adherence increased over time and was higher in the intervention group, the intervention did not significantly change the pattern of change over time. The slight decrease in adherence at 6 months compared with 3 months suggests that long-term behavioral maintenance may be affected by decreased motivation, limited self-regulation, or changes in external circumstances. Given the important roles of self-efficacy and social support in the HAPA model, future research with larger sample sizes and longer follow-up is warranted to elucidate the mechanisms of sustained behavior change and to identify strategies for optimizing long-term exercise adherence in patients with CHF.

### 4.2 Impact of HAPA-based exercise rehabilitation on exercise self-efficacy

The findings of this study suggest that the HAPA-based exercise rehabilitation program may increase exercise self-efficacy in CHF patients. Exercise self-efficacy is a person's confidence in their ability to engage in regular exercise, and this is a critical element in sustaining exercise behavior and promoting the ability to manage oneself (21). At baseline, both groups had low exercise self-efficacy, which is consistent with the results of Tianqi (22) and Li Jianxun (23). This may be due to physical decline as a result of disease symptoms (e.g., dyspnea, fatigue), repeated hospitalizations, and poor experiences with exercise. Additionally, patients have many misconceptions about the safety of exercise, thinking that physical activity will trigger acute episodes. Limited social support and long-term sedentary habits further hinder the development of exercise routines, all of which combine to undermine confidence in the ability to exercise. Three months after the intervention, the intervention group had significantly higher exercise self-efficacy than the control group (*P* < 0.001), indicating that the HAPA-based program may be effective in increasing the confidence of patients to exercise. The stage-based interventions of the model may enhance personal beliefs and perceived behavioral control, leading to the improvement of exercise self-efficacy, though the mechanism has not yet been validated using full data analysis. Personalized health education and goal setting help patients understand the benefits of exercise and alleviate concerns about risks of exercise. Gradual exercise plans, with periodic feedback, allow patients to build up a store of successful experiences, which further strengthens self-efficacy. Clinical practitioners can use this intervention to increase confidence, self-efficacy, and ultimately exercise adherence in patients.

### 4.3 Impact of HAPA-based exercise rehabilitation on quality of life

The results of this study suggest that the HAPA-based exercise rehabilitation program may improve the QoL of patients with CHF. At baseline, there were no significant variances between control and intervention groups in total QoL scores or specific domains (physical, emotional and others), indicating similar starting points. After three months of intervention, the intervention group indicated substantially lower scores than the control group in total QoL as well as the scores for all domains (*P* < 0.001), indicating a positive impact of the HAPA-based program. Beyond statistical significance, observed improvements may also be clinically meaningful. On the MLHFQ, the score is higher, the worse the QoL and a decrease in total score indicates improvement. Previous studies have reported a minimal clinically important difference (MCID) for the MLHFQ of 3.59 to 19.14 points (24). In this study, the difference between groups at the three-month time point was 15.66 points, which is within the reported MCID range, suggesting that the improvement in QoL that occurs in response to the intervention is likely to be clinically meaningful. These findings are consistent with results from the study of Zhu Lijuan et al. (25) in glioma radiotherapy patients, which supports the applicability of the HAPA model to different disease contexts. Patients with CHF usually suffer from dyspnea, fatigue, shortness of breath and an accumulation of fluid. Coupled with high morbidity, mortality, and frequent rehospitalizations, these factors contribute to a heavy burden of disease and generally poor QoL (26). The HAPA model addresses these challenges by stage-based interventions. By individual patient needs, goals setting and building up successful experiences, the program boosts self-efficacy. Additionally, staged behavior change strategies encourage adherence to exercise rehabilitation to achieve comprehensive improvements in QoL while reducing physiological symptoms. However, the mechanisms of these effects need to be validated in future research.

The results of this study indicate that the HAPA-based intervention might be effective at improving exercise adherence, self-efficacy and QoL in patients with CHF. However, the underlying mechanisms are mostly theoretical, since empirical evidence is scarce. Since not all the core constructs of the HAPA model were measured, it was not possible to systematically examine the specific roles of these constructs or the pathways through which the intervention exerted its effects. Thus, this study is preliminary evidence for the use of the HAPA model in behavioral interventions for CHF, rather than a comprehensive validation of the mechanisms of the model. Future research should measure a wider variety of HAPA-related constructs and use mediation or path analyses to understand more about the particular contributions of these constructs to intervention outcomes.

In summary, the exercise rehabilitation program based on the HAPA model may promote exercise adherence, self-efficacy, and QoL in patients with CHF. However, several limitations should be noted. First, this was a single centre study with a relatively small sample size. Eligible patients were consecutively enrolled, and no formal *a priori* sample size calculation was performed, which may influence statistical power, representativeness and generalizability of the results. Second, outcome measures were mostly subjective and not based on objective indicators such as the 6 min walk test, cardiopulmonary exercise testing, biomarkers, or readmission rates. Consequently, the study was not able to fully assess the effects of the intervention on the functional status and clinical outcomes of the patients, which limits the strength of the conclusions. Although the intervention was implemented for three months with follow-up up to six months, the long-term sustainability of the behavioural changes is unknown. Exercise adherence was measured mostly by self-reported scales, with post-intervention data gathered mostly by telephone follow-up, which may have affected the results by introducing self-report bias. Participants in the intervention group had greater contact, supervision and encouragement, WeChat-based follow-up, exercise log recording and continuous feedback, which may cause social desirability and outcome reporting bias between groups, and introduce contact and differential reporting biases. Additionally, five patients dropped out and only those who completed the intervention were included in the final analysis, which may have further biased the results. Finally, exercise self-efficacy and QoL were not assessed at the six-month follow-up, prohibiting assessment of their long-term trajectories and limiting a comprehensive assessment of the sustained effects of the intervention.

## 5 Conclusion

This study suggests that a HAPA-based exercise rehabilitation program may contribute to an improvement of exercise adherence, self-efficacy, and QoL in patients with CHF. While the intervention group had overall better outcomes than the control group, these findings should be interpreted with caution because of the small sample size, single-center study design, and use of self-reported measures. Further large-scale, multicenter studies are warranted to validate these findings.

## Data Availability

The original contributions presented in the study are included in the article/Supplementary Material, further inquiries can be directed to the corresponding author/s.
